# Tobacco and marijuana use during the COVID-19 pandemic lockdown among American Indians residing in California and Oklahoma

**DOI:** 10.18332/tid/174819

**Published:** 2023-12-20

**Authors:** Julie H. T. Dang, Sixia Chen, Spencer Hall, Janis E. Campbell, Moon S. Chen, Mark P. Doescher

**Affiliations:** 1 Division of Health Policy and Management, Department of Public Health Sciences, School of Medicine, University of California, Davis, Sacramento, United States; 2 Department of Biostatistics and Epidemiology, Hudson College of Public Health, University of Oklahoma Health Sciences Center, Oklahoma City, United States; 3 Division of Hematology and Oncology, Department of Internal Medicine, School of Medicine, University of California, Davis, Sacramento, United States; 4 Department of Family and Preventive Medicine, College of Medicine, University of Oklahoma College of Health Science Center, Oklahoma City, United States; 5 Stephenson Cancer Center, Oklahoma City, United States

**Keywords:** smoking, American Indians, tobacco use, marijuana use, COVID-19

## Abstract

**INTRODUCTION:**

American Indian (AI) people experience a disproportionate tobacco and marijuana burden which may have been exacerbated by the COVID-19 pandemic. Little is known about the tobacco and marijuana habits of American Indian individuals during the COVID-19 pandemic. The objective of this study is to examine tobacco and marijuana use as well as change in use during the COVID-19 pandemic among the American Indian community.

**METHODS:**

This cross-sectional study analyzes survey data from a convenience sample of American Indian individuals residing in California and Oklahoma and included adults with and without cancer that resided in both rural and urban areas (n=1068).

**RESULTS:**

During October 2020 – January 2021, 36.0% of participants reported current use of tobacco products, 9.9% reported current use of marijuana products, and 23.7% reported increased use of tobacco and/or marijuana in the past 30 days, with no difference between those with cancer and those without cancer. Tobacco use was associated with marital status, age, employment status, COVID-19 exposure, COVID-19 beliefs, and alcohol consumption. Marijuana use was associated with COVID-19 beliefs, alcohol consumption, and income level. Increased tobacco and/or marijuana use was associated with baseline use of those products. Nearly a quarter of participants reported increased use of tobacco and/or marijuana products during the COVID-19 pandemic.

**CONCLUSIONS:**

We observed high rates of tobacco use during the COVID-19 pandemic, consistent with other studies. Research is needed to examine whether tobacco and marijuana use will decrease to pre-pandemic levels post-pandemic or if these behaviors will persist post-pandemic. Given these findings, there is a pressing need to increase access to evidence-based tobacco and marijuana treatment services in the AI population post COVID-19 pandemic.

## INTRODUCTION

Pre-pandemic, the Centers for Disease Control and Prevention reported that 25.3% of American Indian and Alaskan Native (AI/AN) adults are current cigarette smokers, compared with 12.5% of the general population^1^. This is the highest smoking rate of any US racial or ethnic group. In a study using 2016 to 2019 data from the Behavioral Risk Factor Survey, Jeffers et al.^2^ reported that nearly 15% of AI/AN adults reported marijuana use in the past 30 days. This was the highest marijuana use prevalence of any major racial or ethnic group in the US^2^. The study also found that 7.2% of AI/AN marijuana users were more likely to be daily users, which also was the highest proportion of any racial or ethnic group in this study. The burden of commercial tobacco use in AI/AN communities is well documented in the literature^3,4^. Compared to other racial and ethnic groups AI/AN people have a higher risk of dying from and developing diseases caused by using commercial tobacco products^3,4^.

The COVID-19 pandemic has further exacerbated the already disproportionate health inequities faced by the AI/AN community^5,6^. Researchers found that compared with Black, Latino and White American populations, AI/AN populations have a higher prevalence of socioeconomic and health-related COVID-19 risk factors^7^. AI/AN people are also at higher risk for COVID-19 compared to non-Hispanic Whites^8,9^. Studies suggest shared transportation, household size, and limited access to running water as factors that may contribute to COVID-19 transmission among tribal communities^10,11^. Substance use has been reported as a mechanism to cope with stress and many studies have reported the impact of the COVID-19 pandemic on tobacco and marijuana use^12-15^. Using data from the National Interview Survey, Cornelius et al.^16^ estimated that the number of smokers among AI/AN increased by 110000 from 2011 and 2020. This is the highest reported use of tobacco products among all racial and ethnic groups. Given the disparities in tobacco product use among AI/AN individuals and current studies that have reported an increase to tobacco usage in AI/AN individuals, it is important to not only assess whether the COVID-19 pandemic has increased tobacco and marijuana consumption in this community, but also identify factors associated with these behaviors.

A better understanding of the prevalence of tobacco and marijuana use in AI/AN populations is important not only for designing targeted outreach and education, but also to assess whether additional resources are needed to reduce the tobacco burden created by the COVID-19 pandemic in this community. Thus this cross-sectional survey aims to examine tobacco and marijuana use among AI/AN persons during the COVID-19 pandemic and their correlates using data collected in 2020–2021 from California (CA) and Oklahoma (OK), the two states that have the largest AI/AN populations in the US^17^.

## METHODS

### Study sample

The impact of COVID-19 on the Cancer Continuum Consortium (IC-4), was established to develop and agree upon a set of core survey questions that would be administered by seventeen National Cancer Institute (NCI)-Designated Cancer Centers (Centers) across the US. The purpose of the survey was to assess the impact of COVID-19 on cancer prevention, control, and survivorship. This study reports on the responses from a subset of the survey questions administered by the Cancer Centers at the University of California, Davis and the University of Oklahoma. The surveys conducted by these Centers focused on recruiting participants from the American Indian (AI) communities in their states. The term ‘AI’ is henceforth used throughout the remainder of the article in reference to our sample population rather than ‘AI/AN’, as few Alaska Natives reside in these locations. Both Centers used a cross-sectional, non-probability-based study design for the survey.

### Recruitment

A convenience sample of adult AI individuals were recruited from both Centers. Eligibility criteria included being aged ≥18 years and self-identifying as AI persons. To ensure we had a diverse representation of AI individuals, we recruited persons with and without a history of cancer (n=1068) and those residing in both non-metropolitan (28.5%) and metropolitan (71.5%) areas. AI individuals who resided in counties with Rural-Urban Continuum Codes (RUCC) 1-3 were classified as living in a metropolitan area and AI individuals who resided in counties with RUCC 4-9 were classified as living in a non-metropolitan area^18^. University of Oklahoma recruited both healthy participants and individuals with cancer. Individuals with cancer were recruited through the Oncology Research Information Exchange Network (ORIEN) and the University of Oklahoma cancer registry which consists of adults with a cancer diagnosis within the past five years. Healthy individuals were recruited through digital ads on social media, past research participants who agreed to be recontacted for future research, and from tribal partner advertising (n=657). The University of California utilized two partnering tribal health centers to recruit and administer the survey to their patients (n=411). These participants were offered the survey during scheduled clinic visits and during vaccination clinics for influenza and COVID-19. For both University of California, Davis and University of Oklahoma participants, surveys took roughly 20 minutes to complete and were administered online through a survey link (using a provided IPAD or the participants phone), through phone interviews (with the interviewer recording the answers via the survey link and/or paper survey), and in person (using paper surveys). All survey questions were self-reported and completed by the participant. University of Oklahoma utilized fraud detection tools to prevent false and multiple responses. Survey responses had to originate from the landing page, only one response per IP address was allowed, and research staff manually checked for duplicate emails and responses to open-ended questions that did not make sense. Participants from University of California, Davis, completed the survey in the presence of a research team member. All data were collected and managed using the Research Electronic Data Capture (REDCap) secure web application^19^. Participants who completed the survey through University of Oklahoma received a $5 Walmart gift card and participants from University of California, Davis received a $10 gift card as compensation for their time.

### Measures

The survey consisted of 58 core questions divided into four sections regarding: 1) sociodemographics; 2) health behaviors and status (e.g. diet, exercise, tobacco use, marijuana use, alcohol use); 3) cancer (e.g. screenings, care, treatment, vaccination); and 4) COVID-19 attitudes and behaviors (e.g. testing status, adherence to social distancing recommendations, constraints, support). This study reports on the 24 questions related to sociodemographic, COVID-19-related attitudes and behaviors, and tobacco and marijuana use. See the Supplementary file for survey items.

*Independent measures*

Sociodemographic questions include age group (18–39; 40–59; ≥60 years), sex (female; male), marital status (divided into three categories: single or never married; married or living together; separated, divorced or widowed), minors residing in household (yes; no), Hispanic ethnicity (yes; no), education level (high school diploma/GED or less; some college or more), income level (<$35000; ≥$35000), health insurance status (yes; no), and employment status before COVID-19 (full- or part-time employment; unemployed or other occupation). The other occupational categories included students, retired individuals, homemakers, or disabled. A general health status question (excellent to good; fair to poor) was also included. The education level, income level, health insurance status, and general health status measures were dichotomized during analyses to ensure statistical power and to have adequate sample size.

COVID-19 questions included ever being tested, outcome of test, close physical contact with a COVID-positive person, perceived importance of social distancing recommendations, adherence to social distancing recommendations, and receipt of support (e.g. emotional, materials, financial) during the lockdown.

*Dependent measures*

Participants were given a list of 14 products containing tobacco and/or marijuana and were asked if they have used any of those products in the past 30 days. These were classified as any past 30-day use of tobacco (yes; no) and any past 30-day use of marijuana (yes; no). Also, respondents were asked whether they changed their frequency of tobacco or marijuana use in the past 30 days compared to before the COVID-19 pandemic. This was categorized as any change (more, less, or no change) in past 30-day use of tobacco or marijuana in the descriptive statistics and categorized as increase in both or either product in the bivariate and multivariable analyses (yes; no).

### Statistical analysis

Descriptive statistics including frequencies and percentages were calculated for categorical variables. Binary association between covariate variables (e.g. all variables described in independent measures section) and outcome variables (e.g. tobacco use, marijuana use, and change in tobacco and/or marijuana use) were examined by using chi-squared test or Fisher’s exact test. Multivariable analysis between outcome variables and covariate variables was conducted using multivariable logistic regression. Collinearity and interactions were examined in building the final model. Covariate variables included in the final model for each outcome variable are described below. Adjusted odds ratios were obtained for the association between dependent (i.e. outcome) variables and independent (i.e. ‘predictor’) variables. All tests are two-sided with significance level 0.05. Respondents with missing outcome values were excluded from bivariate and multivariable analysis (missing rates were ≤6% for all predictor variables). All statistical analysis was conducted in SAS® 9.4^20^.

## RESULTS

The overall sample comprised 1068 AI adults (Table 1). The majority of participants were female (62.9%), between the ages of 18–39 years (38.8%), non-Hispanic (81.1%), pursued some post-secondary education (68.2%), had a household income level of ≥$35000 (57.8%), were covered by health insurance or other kind of health plan (83.7%), and were in excellent to good health (71.3%). Most of the participants were married or living with a partner (60.0%), employed full- or part-time before the COVID-19 pandemic (59.4%), and did not have a minor residing in the household (57.5%). At the time of the survey, 75.5% of respondents had not been exposed to COVID-19; 86.9% followed two or more COVID-19 social distancing guidelines; 84.9% believed social distancing guidelines are very to somewhat important, 66.6% were tested for COVID-19; 75.3% had not been diagnosed with COVID-19; and 57.9% did not attend any gatherings with two or people individuals outside of their household. Among participants, in the last 30 days, 36.0% reported using tobacco products and 9.3% reported using any form of marijuana. Of those who reported using tobacco and/or marijuana products, 23.7% reported using these products more frequently during the past 30 days compared to before the COVID-19 pandemic.

**Table 1:** Demographic characteristics and COVID-19 safety behaviors and attitudes of participants (N=1068)

*Characteristics*	*n (%)^a^*
**State**	
OK	657 (61.5)
CA	411 (38.5)
**Age** (years)	
18–39	401 (38.8)
40–59	314 (30.4)
≥60	318 (30.8)
**Sex**	
Female	666 (62.9)
Male	393 (37.1)
**Hispanic**	
Yes	180 (18.9)
No	773 (81.1)
**Education level**	
High school diploma/GED or lower	336 (31.8)
Some college or higher	719 (68.2)
**Income level** ($)	
<35000	405 (42.2)
≥35000	554 (57.8)
**Health insurance**	
Yes	871 (83.7)
No	170 (16.3)
**Health status**	
Excellent to good	751 (71.3)
Fair to poor	303 (28.7)
**Marital status**	
Single/never married	161 (15.3)
Married/living together	631 (60.0)
Separated/divorced/widowed	260 (24.7)
**Employment pre-pandemic**	
Full- or part-time employment	622 (59.4)
Unemployed or other occupation	426 (40.6)
**Minor in household**	
Yes	454 (42.5)
No	614 (57.5)
**Exposure to COVID-19**	
Yes	239 (24.5)
No	737 (75.5)
**Number of social distancing guidelines followed**	
≤2	129 (13.1)
≥3	853 (86.9)
**Importance of social distance**	
Very to somewhat	881 (84.9)
Little to not	157 (15.1)
**Tested for COVID-19**	
Yes	699 (66.6)
No	350 (33.4)
**Diagnosed with COVID-19**	
Yes	173 (24.7)
No	526 (75.3)
**Attend gathering** (≥2 people)	
Yes	450 (42.1)
No	618 (57.9)
**Used any form of tobacco and/or marijuana products in last 30 days**	
Yes	384 (36.0)
No	684 (64.0)
**Used any form of marijuana in the last 30 days**	
Yes	99 (9.3)
No	969 (90.7)
**Change in tobacco and/or marijuana use during the past 30 days compared to before the COVID-19 pandemic**	
More in the past 30 days	116 (23.7)
Less in the past 30 days	171 (35.0)
Same amount in the past 30 days	202 (41.3)

a Percentages may not total 100% because of missing values.

Table 2 shows the characteristics of those who reported having used tobacco and marijuana products and bivariate analyses. Statistically significant measures associated with tobacco use included age group (p<0.0001), sex (p=0.0005), being Hispanic (p=0.0001), health insurance coverage (p<0.0001), marital status (p<0.0001), being employed prior to the COVID-19 pandemic (p<0.0001), belief in the importance of social distancing guidelines (p<0.0001), receiving social support during the COVID-19 pandemic (p=0.011), being exposed to COVID-19 (p<0.0001), being tested for COVID-19 (p=0.0016), being diagnosed with COVID-19 (p=0.0116), attending gatherings (p=0.0301), and living with a minor (p=0.0001). Statistically significant measures associated with marijuana use included age group (p=0.0149), income level (p=0.0173), belief in the importance of social distancing guidelines (p<0.0001) and receiving social support during the COVID-19 pandemic (p=0.0041). The only statistically significant measures associated with an increase in past 30-day use of tobacco, marijuana, or both, was the use of those substance at baseline (p=0.0041).

**Table 2:** Demographic characteristics and COVID-19 safety behaviors and attitudes among tobacco and marijuana users and bivariate analysis

*Characteristics*	*Used tobacco (N=384)*		*Used marijuana (N=99)*		*Increased tobacco and/or marijuana use (N=116)*	
	*n (%)*	*p*	*n (%)*	*p*	*n (%)*	*p*
**Age** (years)		**<0.0001**		**0.0149**		0.0992
18–39	227 (60.7)		48 (50.0)		66 (58.9)	
40–59	86 (23.0)		30 (31.3)		32 (30.4)	
≥ 60	61 (16.3)		18 (18.7)		12 (10.7)	
**Sex**		**0.0005**		0.8717		0.7241
Female	212 (55.9)		63 (63.6)		64 (56.1)	
Male	167 (44.1)		36 (36.4)		50 (43.9)	
**Hispanic**		**0.0001**		0.1201		0.1599
Yes	91 (25.1)		24 (24.7)		33 (29.2)	
No	272 (74.9)		73 (75.3)		80 (70.8)	
**Educational level**		0.1676		0.5082		0.1246
≤High school/GED	269 (67.0)		69 (71.1)		87 (75.7)	
≥College/college degree	111 (29.2)		28 (28.9)		28 (24.3)	
**Income level** ($)		0.5025		**0.0173**		0.0709
<35000	157 (43.6)		51 (53.7)		43 (39.5)	
≥35000	203 (56.4)		44 (46.3)		66 (60.6)	
**Health insurance coverage**		**<0.0001**		0.2735		0.8221
Yes	286 (77.1)		78 (79.6)		87 (78.4)	
No	85 (22.9)		20 (20.4)		24 (21.6)	
**Health status**		0.0643		0.6186		0.3745
Excellent to good	257 (67.8)		67 (69.1)		75 (65.8)	
Fair to poor	122 (32.2)		30 (30.9)		39 (34.2)	
**Marital status**		**<0.0001**		0.119		0.9055
SNM	54 (14.2)		22 (22.5)		17 (14.8)	
MLT	259 (68.2)		54 (55.1)		77 (67.0)	
SDW	67 (17.6)		22 (22.4)		21 (18.3)	
**Employed pre-pandemic**		**<0.0001**		0.2735		0.3632
F/PT	260 (69.0)		62 (63.6)		85 (73.3)	
U/O	117 (31.0)		34 (35.4)		31 (26.7)	
**Exposure to COVID-19**		**<0.0001**		0.4462		0.1483
Yes	128 (36.1)		25 (27.8)		41 (40.2)	
No	227 (63.9)		65 (72.2)		61 (59.8)	
**Number of SD guidelines followed**		0.2324		0.1875		0.513
≤2	50 (38.8)		16 (12.4)		15 (24.6)	
≥3	285 (33.4)		75 (8.8)		87 (28.7)	
**Importance of SD**		**<0.0001**		**<0.0001**		0.9476
Very to somewhat	280 (74.3)		68 (70.8)		86 (74.1)	
A little to not	97 (25.7)		28 (29.2)		30 (25.9)	
**Social support during COVID-19**		**0.011**		**0.0041**		0.2386
Frequently	131 (35.9)		41 (44.1)		45 (40.9)	
Rarely	234 (64.1)		52 (55.9)		65 (59.1)	
**Tested for COVID-19**		**0.0016**		0.3262		0.828
Yes	275 (72.8)		59 (62.1)		81 (73.0)	
No	103 (27.2)		36 (37.9)		30 (27.0)	
**Diagnosed with COVID-19**		**0.0116**		0.4497		0.1248
Yes	54 (19.6)		17 (28.8)		21 (25.9)	
No	221 (80.4)		42 (71.2)		60 (74.1)	
**Attended gatherings** (≥2 people)		**0.0301**		0.3138		0.4349
Yes	239 (62.2)		62 (62.6)		70 (60.3)	
No	145 (37.8)		37 (37.4)		46 (39.7)	
**Minor residing in household**		**0.0001**		0.5337		0.658
Yes	193 (50.3)		45 (45.5)		58 (50.0)	
No	191 (49.7)		54 (54.5)		58 (50.0)	
**Tobacco and marijuana use**						**0.0041**
Dual use	-		-		25 (45.5)	
Tobacco use only					81 (25.2)	
Marijuana use only					7 (19.4)	

MLT: married/living together. SDW: separated/divorced/widowed. SNM: single/never married. F/PT: full- or part-time; U/O: unemployed or other. SD: social distancing. Significant at p<0.05.

In multivariable analyses, characteristics independently associated with higher odds of tobacco use included: being married and/or living together (MLT) compared to those who reported being single, never married (SNM) (OR=1.95; 95% CI: 1.16–3.27); being separated, divorced, or widowed (SDW) compared to being SNM (OR=2.32; 95% CI: 1.26–4.28); being exposed to COVID-19 (OR=1.71; 95% CI: 1.17–2.50); believing that COVID-19 is not important (OR=2.27; 95% CI: 1.47–3.51); and consuming alcohol on two or more days in the past 30 days (OR=4.47; 95% CI: 3.15–6.36). Participants aged 40–59 years (OR=0.30; 95% CI: 0.20–0.45) and participants aged ≥60 years (OR=0.22; 95% CI: 0.13–0.36) had lower odds of tobacco use compared to participants who were aged 18–39 years. Participants who reported having an income of ≥$35000 had lower odds of tobacco use (OR=0.64; 95% CI: 0.44–0.92). Characteristics independently associated with higher odds of marijuana use included believing that COVID-19 is not important (OR=2.69; 95% CI: 1.52–4.74), and consuming alcohol on two or more days in the past 30 days (OR=3.87; 95% CI: 2.28–6.56). Those who reported having an income of ≥$35000 had lower odds of using marijuana (OR=0.50; 95% CI: 0.30–0.84). Participants who reported using both tobacco and marijuana compared to using tobacco alone (OR=2.81; 95% CI: 1.44–5.61) had higher odds of increased tobacco and/or marijuana use. When we removed this variable from the model, only age was significantly associated with increased tobacco and/or marijuana use (Table 3).

**Table 3:** Multivariable regression with predictors of tobacco, marijuana, and increase in tobacco and/or marijuana use

*Variables*	*Tobacco use (N=834)*	*Marijuana use (N=834)*	*Increased tobacco and marijuana use (N=348)*
	*OR (95% CI)*	*OR (95% CI)*	*OR (95% CI)*
**Age** (years)			
40–59 vs 18–39	0.30 (0.20–0.45)***	1.25 (0.67–2.32)	1 (0.50–1.96)
≥60 vs 18–39	0.22 (0.13–0.36)***	0.96 (0.44–2.10)	0.46 (0.16–1.29)
**Marital status**			
MLT vs SNM	1.95 (1.16–3.27)**	0.65 (0.32–1.32)	1.43 (0.59–3.44)
SDW vs SNM	2.32 (1.26–4.28)**	1.04 (0.46–2.38)	1.90 (0.64–5.62)
**Employment status**			
F/PT vs U/O	0.82 (0.54–1.24)	1.06 (0.57–1.97)	1.19 (0.60–2.38)
**Income level** ($)			
≥35000 vs <35000	0.64 (0.44–0.92)*	0.50 (0.30–0.84)**	1.17 (0.68–2.00)
**Exposure to COVID-19**			
Yes vs No	1.71 (1.17–2.50)**	1.03 (0.59–1.80)	1.29 (0.76–2.19)
**Importance of social distancing**			
Not important vs	2.27 (1.47–3.51)***	2.69 (1.52–4.74)***	0.87 (0.49–1.57)
Important			
**Consumed alcohol ≤2 days**			
Yes vs No	4.47 (3.15–6.36)***	3.87 (2.28–6.56)***	0.85 (0.49–1.48)
**Tobacco and marijuana use**			
Dual use vs marijuana only	-	-	2.89 (0.99–8.39)
Dual use vs tobacco only			2.81 (1.41–5.61)**

MLT: married/living together; SDW: separated/divorced/widowed. SNM: single/never married; F/PT: full- or part-time; U/O: unemployed or other. Boldface indicates statistical significance.

*** p<0.001,

** p<0.01,

* p<0.05.

## DISCUSSION

To our knowledge, this is the first and largest study to examine both tobacco and marijuana use among AI adults with and without a history of cancer in two states during the COVID-19 pandemic. While about a third of participants decreased their tobacco and/or marijuana use (35%) and a little less than half did not change their use patterns (41%), we observed higher prevalence of self-reported tobacco use and a higher increase in tobacco and/or marijuana use during the pandemic in this sample of AI adults compared to other studies^16,21,22^. Our results are similar to a systematic review of the impact of COVID-19 on smoking which reported both increases and decreases in tobacco consumption habits^23^. Decreases in tobacco and/or marijuana use may be linked to fears of having COVID-19 complications due to smoking^24^ and having less opportunities to socialize because of the lockdown measures. Increases in tobacco and/or marijuana use can be attributable to COVID-19 induced stress^25^. More research is needed assess the long-term effects of the COVID-19 pandemic on tobacco and/or marijuana consumption in AI/AN communities.

In our study population, the prevalence of tobacco use in the past 30 days during COVID-19 was 36%. This is higher than what was reported by Gaffney et al.^21^, which estimated AI/AN persons smoking prevalence to be 25–27% in 2020^21^ and the Centers for Disease Control (CDC) which reported in 2020, 34.9% of AI/AN individuals used tobacco products^16^. The difference between our sample and those reported by others may be explained by geography, as Gómez-Gómez and Almeda^23^ also reported varied tobacco consumption patterns by US region/state. The prevalence of marijuana use in the past 30 days during COVID-19 in our sample was 9.3%. We found no other study that reports marijuana use among AI/AN adults during COVID-19; however, Brenneke et al.^24^ reported the overall percentages of US adults using marijuana amid the COVID-19 pandemic ranged from 9.2% to 11.3%. Our sample is aligned with their findings, although their study does not disaggregate AI/AN adults.

Similar to the CDC report, we found that tobacco use increased during the COVID-19 pandemic. The CDC reported a 5.6% increase in tobacco product use among AI/AN individuals from 2019 to 2020, but our study found that 23.7% of AI adults tobacco and marijuana users reported increasing their product use in the past 30 days^16^. Chen et al.^26^ found an increase of 9.0% in smoking among the general US adult population during the pandemic. Although the wording in our survey combined any change in tobacco use or marijuana use as one question, the percentage of AI adults in our sample reporting increase use is concerning. More research is needed to assess whether the increase in usage in our sample is attributable to an increase in tobacco use or an increase in marijuana use. Nevertheless, the increases in tobacco and/or marijuana use is likely to have long-term adverse health effects. Nationally, the prevalence of COPD among AI/AN populations is 11.9%, representing the highest rate among all race/ethnicity categories^22^. Additionally, lung cancer mortality rates in CA and OK for AI/AN populations represent some of the highest rates for each state, respectively. In CA, the mortality rate is 56.5/100000 compared to 27.1/100000 for Non-Hispanic Whites and in OK the rate is 70.5/100000 compared to 50.4/100000 for Non-Hispanic Whites^27,28^. Research is needed to see if tobacco and/or marijuana use rates will decrease post-pandemic to pre-pandemic levels, or if these behaviors will persist.

### Limitations

This study has several limitations. First, this is a convenience, cross-sectional sample with participants self-reporting tobacco and marijuana use. There is a potential for unmeasured confounding, thus conclusions about causality should be considered carefully when interpreting the findings of our study. While the OU sample recruited participants with and without a history of cancer, we do not have any additional cancer related information on the individuals with cancer. More detailed surveys are needed to explore the impact of COVID-19 on AI participants with a history of cancer. Additionally, we were not able to disentangle medical marijuana use from recreational use in our survey. In OK, 9.3% of adults use medical marijuana and in CA 4.9% of adults reported marijuana use^29^. Furthermore, the respondents in our sample had on average a higher education level and higher household income than the general population of AI persons in CA and OK^30^. Also, participants who selected Indian Health Services (IHS) as their health coverage were included in the group having health insurance or some other kind of healthcare plan (n=21). As IHS eligibility is not equivalent to having health insurance or a health plan, this could have resulted in these respondents reporting they had coverage when they, in fact, lacked it. Our study was exploratory, thus future studies should include theory-informed measures aimed at exploring underlying mechanism leading to tobacco and marijuana use (e.g. age at first use, level of addiction, and peer influence). Follow-up studies should also assess the frequency of increase, dose of increase, and assess tobacco and marijuana product use separately. Additionally, the findings of this study are limited to AI communities and may not be generalizable to other ethnic groups and countries.

Despite these limitations, this study has several strengths including being one of the first studies to examine the impact of COVID-19 on tobacco and marijuana use among AI adults, having a large and diverse sample of AI adults from two states, and the recruitment of participants with and without cancer from both metropolitan and non-metropolitan areas.

## CONCLUSIONS

In this unique AI sample, we observed high rates of tobacco use during the COVID-19 pandemic, consistent with other studies of the population. Furthermore, nearly one-quarter of participants reported increasing their tobacco and or marijuana use in the past 30 days. COVID-19 placed an immediate burden on AI/AN communities in terms of mortality and morbidity^11,31^ and studies have shown that individuals with tobacco use history were more likely to require hospitalization or die from COVID-19^32,33^. Unless the increase in rates seen here are reversed quickly, the pandemic may have placed an additional long-term health burden on AI people in OK and CA, because downstream increases in tobacco and marijuana-related mortality and morbidity would be expected to occur. Given these findings, there is pressing need to increase access to evidence-based tobacco and marijuana treatment services in the AI population post COVID-19 pandemic.

## Data Availability

The data supporting this research cannot be made available for privacy or other reasons.
