# Supplementary material for: Tobacco and marijuana use during the COVID-19 pandemic lockdown among American Indians residing in California and Oklahoma
Source: Tob Induc Dis. 2023 Dec 20;21:171. doi: 10.18332/tid/174819 (PMC10731663; doi:10.18332/tid/174819)
Supplement: Supplementary file 1 [file TID-21-171-s1.pdf]

1. Have you been tested for COVID-19 by a medical doctor or other healthcare professional?

- Yes, I was tested, and it showed that I had/have COVID-19
- Yes, I was tested, and it showed I did NOT have COVID-19
- Yes, I was tested, and I am waiting for the results
- No, I tried to get tested but could not get a test
- No, I have not tried to get tested
- Don’t know
- Prefer not to answer

2. In the past 30 days, have you been in close physical contact with a person who has tested positive for COVID-19?
    - Yes
    - No
    - Don’t know
    - Prefer not to answer

In an effort to reduce the spread of COVID-19, many are practicing social distancing. Social distancing, also called physical distancing, means keeping space between yourself and other people outside of your home.

Since March 2020, what types of social distancing are you doing all or most of the time?

3a. Staying at home except for going to work, outdoors to exercise, or going to the grocery store, pharmacy, or to get medical care?

- Yes
- No
- Don’t know/not sure

3b. Not having relatives, friends, or neighbors come into your home?

- Yes
- No
- Don’t know/not sure

3c. Staying 6 feet away from people when you leave your home?

- Yes
- No
- Don’t know/not sure

3d. Wearing a face covering/mask when you are outdoors?

- Yes
- No
- Don’t know/not sure

3e. Wearing a face covering/mask when you are inside a store or other place besides your home?

- Yes
- No
- Don’t know/not sure

4. Since March 2020, when COVID-19 restrictions began, have you attended the following?

a. Any gatherings, not including work, with more than 2 people who do not live in the same house as you?

- Yes
- No

b. A rally or demonstration of 20 or more people?

- Yes
- No

c. Other large-scale social gatherings of 20 or more people?

- Yes
- No

5. How important do you think social distancing is during COVID-19?

- Very important
- Somewhat important
- A little important
- Not important
- Don’t know/not sure

6. In the past 2 weeks, how often have you received support (e.g., emotional, materials, or financial support) from friends or loved ones to help you during the COVID-19 pandemic?

- Every day
- Several times a week
- Once a week
- Once in 2 weeks
- Never
- Don’t know/not sure

7. In general, how would you say that your health is? Excellent

- Very good
- Good
- Fair
- Poor
- Don’t know/not sure

8. During the past 30 days, have you used any of the following tobacco and/or marijuana products?

- Cigarettes
- Little cigars
- Cigarillos (e.g., Black & Mild)
- Hand-rolled cigarettes
- Cigars (without marijuana)
- Blunts (without marijuana)
- Marijuana (rolled in paper)
- Pipe
- Bidi
- Smokeless tobacco or dip
- Electronic cigarettes containing nicotine
- Electronic cigarettes containing marijuana

- Hookah or waterpipe
- Other products (please specify) ____
- I have not used any tobacco products in the past 30 days (*Go to question 24*)

9. Have you changed the frequency of tobacco and/or marijuana use compared to BEFORE the COVID-19 pandemic?

- Yes, I have used tobacco and/or marijuana products MORE compared to before the pandemic
- Yes, I have used tobacco and/or marijuana products LESS compared to before the pandemic
- No, I have been using the SAME amount of tobacco and/or marijuana products compared to before the pandemic
- Don't know/not sure

10. What is your date of birth? (MM/DD/YYYY)

11. What County do you live in?

12. What is your zip code?

13. What is your sex?

- Male
- Female
- Prefer not to answer

14. Are you of Hispanic origin?

- Yes
- No
- Prefer not to answer

15. What is your race? (mark all that apply)

- White
- Black or African American
- American Indian or Alaskan Native
- Asian or Asian American
- Native Hawaiian or other Pacific Islander
- Arab
- Other (specify)___________
- Prefer not to answer

16. What is the highest grade or level of school you completed?

- Less than high school
- Some high school, no diploma
- GED
- High school graduate
- Some college but no degree
- Associate degree-occupational/vocational
- Associate degree-academic program
- Bachelor's degree (e.g., BA, AB, BS)
- Master's degree (e.g., MA, MS, Meng, Med, MSW)

- Professional school degree (e.g., MD, DDS, DVM, JD)
- Doctorate degree (e.g., PhD, EdD)
- Prefer not to answer

17. Which category best describes your occupational status in February 2020 prior to the stay-at-home orders put in place as a result of the COVID-19 pandemic? (mark all that applies)

- Employed Full-time
- Employed Part-time
- Unemployed
- Homemaker
- Student
- Retired
- Disabled
- Other (specify)______
- Prefer not to answer

18. Are you currently being paid for a full or part-time job, including being paid by a job while you stay home? Do not include unemployment compensation.

- Yes
- No
- Prefer not to answer

19. Thinking about members of your family living in your household, what is the combined annual income, meaning the total pre-tax income from all sources earned in the past year?

- $0 to $9,999
- $10,000 to $14,999
- $15,000 to $19,999
- $20,000 to $34,999
- $35,000 to $49,999
- $50,000 to $74,999
- $75,000 to $99,999
- $100,000 to $199,999
- $200,000 or more
- Prefer not to answer

20. What is your current marital status?

- Single, never been married
- Married
- Not married but living together
- Separated
- Divorced
- Widowed
- Other (specify)_______
- Prefer not to answer

21. Are you covered by health insurance or some other kind of health care plan?

- Yes
- No (*Go to question 59*)

- Prefer not to answer

22. What type of health insurance do you have? Select all that apply.

- Medicaid
- Private health insurance
- Medicare
- Medicare plus a supplemental policy
- Military/VA
- Other (specify)________
- Prefer not to answer

223. How many adults live in your household including you? ____

24. How many children (less than 18 years of age) live in your household? ____ Please provide the age and sex of each child. If child is less than 1 year old, please enter 1.

21a. Child 1 is ____years of age.

21b. Please provide the sex of child 1.

- Male
- Female
- Prefer not to answer

21c. Child 2 is_____years of age.

21d. Please provide the sex of child 2.

- Male
- Female
- Prefer not to answer

21e. Child 3 is_____years of age.

21f. Please provide the sex of child 3.

- Male
- Female
- Prefer not to answer

© 2023 Dang J.H.T. et al.
